# Spatial Modeling in Environmental and Public Health Research

**DOI:** 10.3390/ijerph7041302

**Published:** 2010-03-26

**Authors:** Michael Jerrett, Sara Gale, Caitlin Kontgis

**Affiliations:** 1 Division of Environmental Health Science, University of California, Berkeley, 710 University Hall (Office and GIS Lab), Berkeley, CA 94720, USA; E-Mail: kontgis@berkeley.edu; 2 Division of Epidemiology, University of California, Berkeley, 710 University Hall (Office and GIS Lab), Berkeley, CA 94720, USA; E-Mail: sagale@berkeley.edu

**Keywords:** GIS, spatial modelling, air pollution, autocorrelation, overlay, spatial regression, remote sensing

## Abstract

This paper has two aims: (1) to summarize various geographic information science methods; and (2) to provide a review of studies that have employed such methods. Though not meant to be a comprehensive review, this paper explains when certain methods are useful in epidemiological studies and also serves as an overview of the growing field of spatial epidemiology.

## 1. Introduction

In this paper, we review the use of Geographic Information Systems (GIS) and spatial analysis in environmental epidemiology and public health research. Spatial epidemiologists, health geographers, and others using geographic methods have made significant contributions to understanding potential exposure pathways in space and time, mechanisms that may influence effective biological dose, modeling of the social distributions of pollutants, and finally the assessment of health effects from environmental contaminants. There has also been considerable attention paid to the perceptions of environmental risk and how this may in turn condition biological responses to pollutants or lifestyle factors such as smoking, which may affect subsequent individual-level susceptibility.

The focus here is on the quantitative aspects of environment risks and how health geographers and others have approached the assessment of risks arising from environmental exposures. Our emphasis is on methods used to study environmental exposures, susceptibilities, ways of adapting, and ultimately the health risks of environmental exposures to human populations. Although we touch upon some of the historical aspects of the use of spatial analysis in public health research, we have drawn specifically on recent research published between 2005 and 2008 to emphasize innovations and emerging trends in the field. Interestingly, this review suggests extraordinarily rapid growth in the use of advanced geographic information science and spatial modeling for addressing questions of environmental risk. The growth in the field has meant that much of the application of spatial analysis has been conducted increasingly by people from disciplines beyond the field of Geography.

To illustrate the utility of specific methods, we draw examples related to environmental justice, atmospheric pollution, and climate change. We aim the paper to a broad audience who may be unfamiliar with epidemiology and spatial analysis; therefore, some technical details are omitted. Numerous references are given on the statistical models for readers interested in operationalizing these methods, as well as specific examples.

## 2. An Operational Framework for Spatial Epidemiology and Public Health

**Figure 1.**
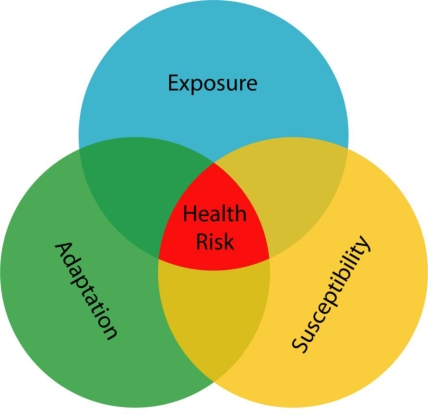
Extended Conceptual Framework for Spatial Analysis in Epidemiology and Public Health (Adapted from Jerrett, Gale and Kontgis, 2009 [3]).

Here we translate Mayer’s [1] conceptualization of health and place into an operational framework that includes three underlying geographies: exposure, susceptibility, and adaptation. In many instances, health geographers have explored single domains, but in others they have sought to understand areas of maximal overlap where two or more of the circles in the Venn diagram (see Figure 1) converge to geographies of risk [2]. The analytic framework we use hinges on four related concepts: (1) geography of susceptibility; (2) geography of exposure; (3), geography of adaptation, and (4) points of intersection between these three, which we call the geography of risk. We discuss how each concept encompasses many lower-level issues such as meteorological dispersion of pollutants, time-space activity patterns, behavioral changes in relation to perceived or real danger, and population distributions of susceptible individuals in time and space. Environmental health geography often focuses on understanding the overlap of two or more of these spheres of influence.

Modeling combines both visualization and exploration techniques, and the statistical analysis assesses whether spatial patterns apparent in the data have occurred by chance or whether they display significant departures from random or control expectation. Spatial modeling usually focuses on data in the following forms: points (e.g., the location of individuals who have died in a given period), point attribute (e.g., estimates of pollution at a fixed-site monitor), areal form (e.g., a census tract polygon with an age-adjusted mortality rate), or continuous surface form (e.g., surfaces of pollution interpolated from estimates of fixed-point attributes). Point pattern maps are referred to as “dot” or “dot density” maps. Areal data maps are called “choropleth” maps. Maps displaying continuous surfaces are usually referred to as “contour”, “isoline” or “isopleth” maps [4,5]. Four processes and associated methods underlie most spatial modeling: autocorrelation tests, interpolation, point pattern analysis, and spatial correlation and regression. Each of these processes is discussed in turn with examples below (portions of the paper have been adapted from Jerrett *et al*. 2003 [6]).

## 3. Spatial Modeling for Public Health

### Overlay Analysis

Overlay analysis is the simplest form of spatial modeling, and consists of stacking different thematic maps on top of one another. This method was employed by Lindley *et al*. [7] to consider conurbation-scale risk and adaptation assessment methods to study the response of the greater Manchester urban area to climate change. This new, explicitly spatial method was developed to address the lack of information needed to adapt to climate change.

**Figure 2.**
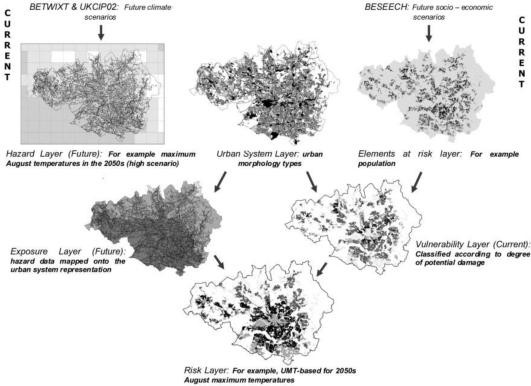
Application of conurbation-scale risk assessment. Lindley *et al*. overlay several different input values for climate change to develop a risk map to show areas most affected by climate change in Manchester, UK.

Conurbation-scale risk assessment was performed to evaluate an entire urban-system as well as provide a basis for neighborhood-level analyses. Similar to the conceptual framework introduced earlier, the authors defined risk to be an interaction between hazard, exposure, and vulnerability. This methodology uses GIS to create separate maps of various risk elements (*i.e.*, population), hazards (*i.e.*, maximum August temperatures), and the urban-system (*i.e.*, urban morphology types). A layer that maps the current vulnerability of the region is then created by merging the risk element layers to the urban-system layer, and a layer that projects future exposure is created by merging the hazard layer to the urban-system layer. Finally, the projected exposure layer and current vulnerability layer are merged to create a final risk layer (see Figure 2).

**Figure 3.**
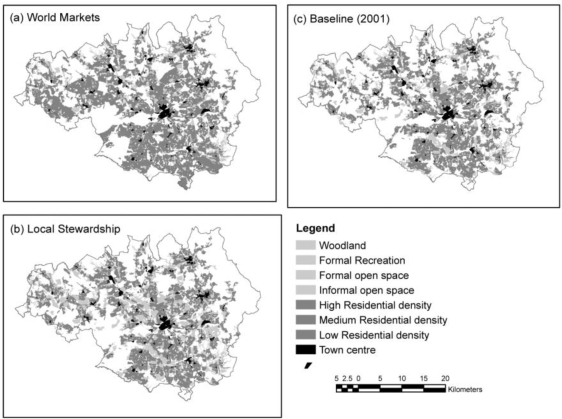
Using conurbation-scale risk assessment to analyze heat stress risk. This figure is adapted from Lindley, *et al*. (2007) to show the projected greenspace and residential characteristics for Manchester, UK in the 2050s in maps (a) and (b). These characteristics are based on different socio-economic scenarios with map (c) representing the baseline in 2001. For more details regarding these methods, refer to Lindley *et al*. (2007).

To demonstrate the method, the authors used conurbation-scale risk assessment to analyze how socio-economic change will affect the risk of heat stress (see Figure 3). This case study led the authors to make several policy suggestions that could help mitigate overall risk to heat stress in the Greater Manchester area, UK. To increase an individual’s personal adaptive capacity, the authors propose longer working lives to provide health coverage and to create stronger social networks. Additionally, the authors recommend urban densification and an improved transport system so that the region can grow without increasing social deprivation. Finally, the authors encourage increased greenspace cover to reduce the heat hazard.

The authors reported this methodology to be valuable for several reasons. Firstly, since each risk element is represented as a separate layer, it is possible to modify each element individually to re-assess the final risk layer. This allows planners to easily evaluate different adaptation strategies to determine how best to mitigate the risk faced by urban areas due to climate change. Secondly, by developing this GIS method it is possible not only to identify current areas where adaptation is most necessary to deal with the risks posed by climate change, but also possible to identify areas that are most at risk in the future. Finally, to perform the conurbation-scale risk assessment, the authors used previously generated data to create the various GIS layers. By using the best available data, it was possible to produce results rapidly, which will become increasingly necessary in order for urban areas to adapt swiftly to climate change.

By employing conurbation-scale risk assessment, the authors demonstrated the usefulness of visualization and cartographic overlay. This assessment is efficient and can be completed relatively quickly since it utilizes the best available spatial data rather than creating new data. It also allows researchers to easily compare various risk scenarios to discern the proper adaptive approach to climate change.

Other research uses overlay analysis to identify areas of environmental justice concern. Environmental justice occurs when a certain social group is disproportionately impacted by harmful land uses. This has become an increasingly important topic in the study of health disparities. Researchers have recently sought answers to the health risks of residential racial segregation. In the paper titled *Separate and Unequal*, authors Morello-Frosch and Jesdale implement a GIS model across the US to examine area-level factors, racial segregation, and estimated cancer risk associated with exposure to ambient air pollution [8]. While poverty can be intertwined with racial segregation, there is an independent relation between racial segregation and disparities in exposure to harmful pollutants. This analysis has expanded the idea of segregation through the exploration of several different racial/ethnic groups and the thoughtful adjustment for factors confounding racial inequality.

### Autocorrelation

First we will discuss methods for assessing autocorrelation among observations. Tobler’s [9] oft-cited first law of geography captures the essence of spatial autocorrelation: “everything is related to everything else, but near things are more related than distant things”. In other words, spatial autocorrelation means attribute values (say mortality) of proximal entities (say metropolitan areas) will likely be more clustered or share similar values than distant ones. This is similar to time series data where we would expect to see mortality rates, for example, that are one day apart to be more similar than mortality rates three months in the future. Although similar, spatial autocorrelation tends to be more complex than serial autocorrelation in time series. First, temporal processes can only move in one direction (*i.e.*, from present to future), whereas spatial processes are two-dimensional (*i.e.*, involve area and direction around a compass). They may also have a third dimension (e.g., the area and depth of a ground water aquifer). Second, the metric used to measure distance can vary (e.g., Euclidian distances or functional distances such as travel time or monetary cost) [10]. Thus the two factors, dimensionality and functional distance, make analysis of spatial autocorrelation more complex to model than its temporal counterpart.

Using ambient air pollution as an example, we might expect pollution levels to be more similar between Pittsburgh and Johnstown (a nearby city) than between Pittsburgh and Seattle. This may occur because of similarities in the underlying social and economic processes that cause pollution (e.g., manufacturing base) or atmospheric processes that suspend pollutants over large distances and disperse pollutants from region to region (e.g., prevailing wind patterns). Usually the level of spatial autocorrelation would diminish as a function of distance between the two regions, unless there is some reason for similarity due to industrial structure or some other factor associated with the pollution phenomenon such as transportation emissions. Autocorrelation tests use point, line, or area features that have attribute values attached to them. One important distinction in these tests is whether they measure global or local autocorrelation.

Global autocorrelation tests measure the tendency, across all data points, for higher (or lower) values to correlate more closely together in space with other higher (or lower) values than would be expected if the data points were drawn from a random distribution. Several tests of global autocorrelation are available, with the Moran’s I being the most common. Positive values of the Moran’s I [4] with significant p-values (*i.e.*, p < 0.05) suggest high values in region i tend to depend on values in adjacent regions j (*i.e.*, higher values will cluster in space with other high values). Negative values would suggest that high values tend to associate with low values, similar to a checker board pattern. To understand how autocorrelation tests work, it is useful to distinguish spatial autocorrelation from ordinary correlation. Autocorrelation is defined for observations lagged in time or space with a single sequenced variable, whereas ordinary correlation refers to the joint observation of two or more variables [11]. In global tests for autocorrelation, it is assumed that the relationship between nearby or otherwise connected observations will remain the same everywhere in the study area (referred to as “stationarity” or “structural stability”). For example, the spatial autocorrelation between mortality rates in metropolitan areas of the United States would be the same at all places in the country, meaning the relationship between the areas was purely a function of distance between the areas and not relative location. A positive autocorrelation suggests that like values tend to be located nearby one another.

**Figure 4.**
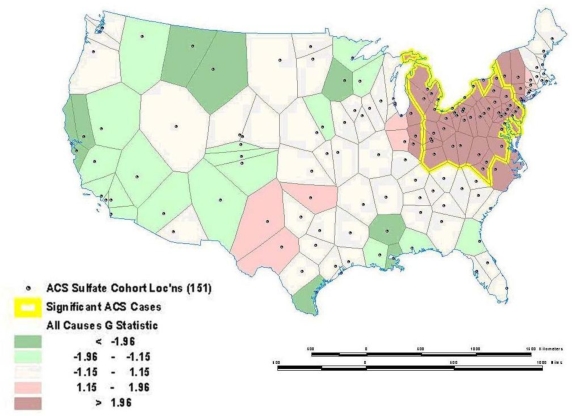
Local Mortality Cluster as Measured by the Getis-Ord Statistic (from ACS cohort). Residual mortality unexplained by 44 individual risk factors (e.g., smoking) with a significant cluster of high residual mortality shown in the darker pink color with the yellow outline as estimated by the Getis-Ord Autocorrelation Statistic.

**Figure 5.**
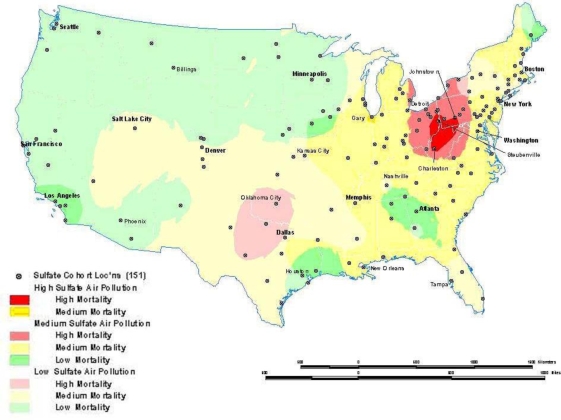
Sulfate Air Pollution and All Cause Mortality Overlay Map (from ACS cohort). Overlay showing intersection between the residual mortality discussed above in Figure 4 and tertiles of sulfate particulate air pollution. The overlay is suggestive of an association between high residual mortality not explained by individual risk factors such as smoking and high air pollution.

Sometimes global relationships are of less interest than local relationships or clusters that may display non-stationarity. Local indicators of spatial association (LISA), such as the local Getis-Ord (G) and local Moran’s I statistics, can assess clustering in small areas to identify clusters or “hot spots” of high or low values (see [121–126] for computational details). These local statistics usually break the study area into smaller regions to determine if local areas have attribute values that are higher or lower than would be expected based on the global average or a random expectation for the entire study area. Using the G statistic to investigate mortality in the American Cancer Society (ACS) Cancer Prevention II Study in 1982 on approximately 550,000 subjects followed for vital status until 1989, we found a significant mortality cluster in the lower Great Lakes area (see Figure 4) [17]. This corresponds roughly to the high mortality-high pollution area shown in Figure 5.

**Figure 6.**
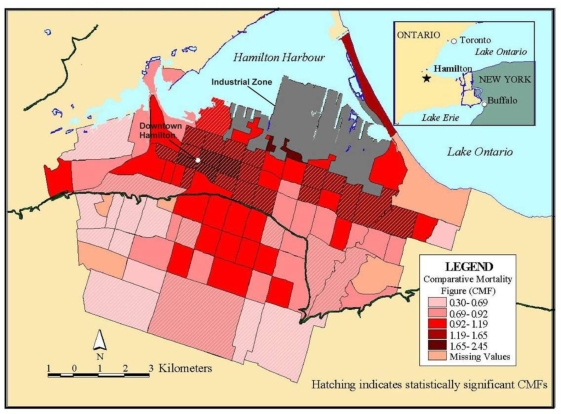
Comparative Mortality Figures for Men Ages 0−74 in Hamilton (1985−94). Comparative mortality figures allow for age standardization using methods similar to a standardized mortality index (see Fleiss 1981 [20] for more detail).

A major issue in the assessment of global or local spatial autocorrelation is the selection of a “spatial weights” or “connectivity” matrix. To assess autocorrelation, it is necessary to assign a matrix that formalizes the potential for spatial dependence. The simplest form of connection is the nearest neighbor approach using a series of polygons such as the census tracts in Figure 6 [18]. With this approach, we would assign a value of one for those neighbors that are connected to each other (*i.e.*, shared a boundary) and a value of zero for those that did not have a connection. The other way to define the weights matrix is with distance. Most distance matrices rely on Euclidian distance, although many modifications are possible if there is prior knowledge about the spatial process in question (e.g., travel time instead of straight line distance). To assign a more complex and realistic spatial weights matrix, it is essential to have prior knowledge about the processes that may have generated the spatial autocorrelation (e.g., population exchanges between cities based on commuting flows may result in a shared exposure to a harmful environmental contaminant and resulting higher mortality rates). Unfortunately, this information is often lacking, so spatial analysts must resort to some arbitrary spatial weight matrix such as the nearest neighbor or Euclidian distance. Other methods are available to assist with selecting an appropriate distance [19]. In the absence of prior information to define the weighting matrix, sensitivity analysis using different weight matrices can be used. If autocorrelation is robust to different assumptions about the connections between places, we have stronger evidence that the results of the autocorrelation tests reflect some real effect and are not an artifact of the assumptions made about spatial dependence in the weight matrix.

Other methods can examine more than one type of event and multiple confounding variables at once, yielding a more informative control for confounding and assessment of autocorrelation. The generalized linear mixed models, generalized additive models (GAM), and Bayesian models are some techniques that allow for adjustment of spatial confounding (e.g., residential clustering by age and race) [4]. As applied to the above example, these approaches would enable the study to assess the effect of pollutant exposure and control for other confounding variables such as age or smoking status.

**Figure 7.**
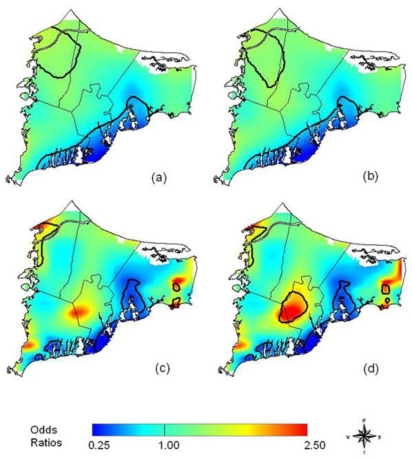
An example of maps created using Generalized Additive Modeling techniques by Webster, *et al*.

In an example from the literature, Webster, *et al*. [21] use a GAM to examine the spatial distribution of breast cancer cases in Cape Cod, MA, and determine if there is clustering of the disease. The GAM was applied to case-control data to minimize spatial confounding as well as the bias that arises from mapping diseases with long latency periods [21]. To map the cancer distribution on Cape Cod, Webster *et al*. condition on several variables including residential history, age and race. Figure 7 illustrates (a) the crude, unadjusted odds ratios (OR) with a span or smooth function of 35% of the data, (b) the adjusted model with race, (c) the crude ORs with a 15% span, and (d) the adjusted model showing spatial confounding by race with a 15% span.

### Interpolation

Interpolation is a process whereby known data points are used to infer values over a space between the points to create a continuous surface. For example, data from a network of pollution monitoring stations may be interpolated to estimate the most likely values between sample locations. There are several different types of interpolation, including kriging, inverse distance weighting, splining and Thiessen polygons [4]. Although such models are often used to predict likely values for exposure assessments, they can also form the basis of visualizing spatially continuous data. For example, Figure 8a for the ACS study shows interpolated concentrations of ambient sulfates from fixed pollution monitors in 151 metropolitan areas of the United States. The high sulfate values in the lower Great Lakes region show a similar spatial distribution to the mortality rates in Figure 6. This pollution surface is the same one used to generate Figure 5. Some limitations to interpolation methods are the smoothing of local trends, the assumption that the surface is continuous, and that there are enough data points to make a valid prediction of the surface (*i.e.*, lower accuracy with fewer data points).

A special type of optimal interpolation known as “kriging” can be used to generate predicted values and their standard errors. These standard errors show where the interpolation tends to be less reliable. Kriging models exploit spatial dependence in the data to develop smoothed surfaces. The spatial dependence can be divided roughly into two broad categories. First-order effects measure broad trends in all the data points such as the global mean, whereas second-order effects measure local variations at shorter distances between the points [4,22]. Kriging models are considered optimal interpolators because they supply the best linear unbiased estimate (BLUE) of the variable’s value at any point in the study area [22]. Figure 8b shows the standard errors of the interpolated sulfate surface of Figure 8a. From this map, we can see that estimates are more reliable in areas where the cities with monitoring stations are denser, especially in the Northeast and Midwestern regions. Errors in the estimates are not often shown with interpolations, and this can lead to incorrect interpretations. In addition, it must also be kept in mind that the data set will determine the results. The sulfate pollution maps shown here depict spatial variation in the ACS sample of 151 metropolitan areas and are not necessarily representative of the spatial pattern that would be found for all the United States in a more complete sample.

### Point Patterns

**Figure 9.**
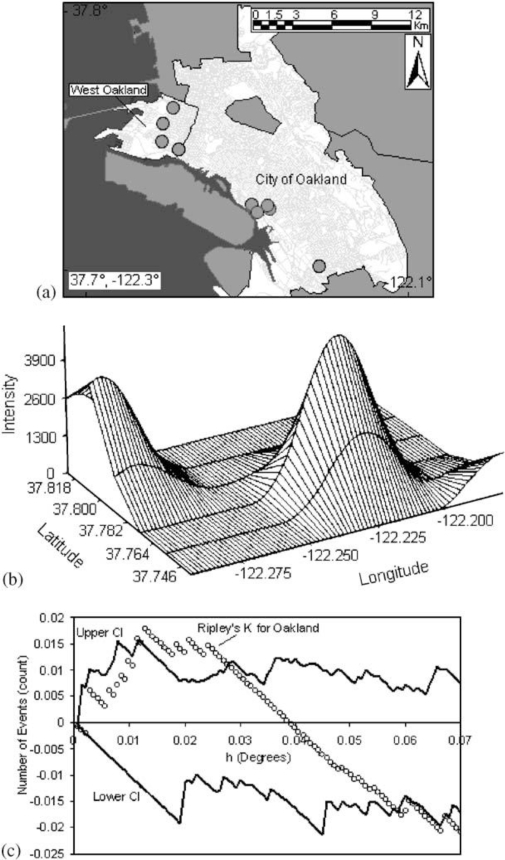
(a) TRI facilities in the city of Oakland, CA (b) Intensity distribution of TRI facilities in the city of Oakland, CA (c) Ripley’s K function for TRI facilities in the city of Oakland.

A third type of modeling deals with the intensity of point patterns over space. This type of modeling addresses the hypothesis that the intensity of point clustering in a given area differs significantly from a random (or control) pattern observed in the entire study area [23,24]. The concern here is with the location and the presence or absence of a disease or condition. For example, we might investigate the clustering of a specific disease known to be linked to an environmental contaminant. This helps identify disease or mortality clusters that may appear in proximity to a pollution source or some other potential risk factor. One major limitation of point pattern models arises from the nature of the data. Point events by definition carry one dimensional information about the event (usually disease or death at that location) which cannot be adjusted easily for other confounding factors such as age. Given the important role age plays in health and survival experience, this is a major shortcoming. Though cluster analyses are an example of the autocorrelation of related points that can display disease patterns as well as exposure patterns and facilitate identification of environmental justice sites. Figure 9, taken from Fisher, *et al*. [25] shows point intensity estimates of Environmental Protection Agency (EPA) designated Toxic Releases Inventory (TRI) facilities in the San Francisco Bay Area. With three different spatial scales—regional, countywide, and citywide—the researchers evaluate the density of TRI sources and whether the clusters are statistically significant with a first-order intensity distribution and a second-order Ripley’s K function. Ripley’s K is a method that compares a given distribution of points to a homogeneous Poisson distribution that is characterized by complete spatial randomness (CSR). Deviations from CSR can then be evaluated for significance. Ripley’s K calculates the relative distance between points by forming circles around randomly chosen points, and estimates the average number of points per unit area. Then the function compares the observed estimate to an expected estimate that is based on CSR.

At the regional level, Fisher *et al*. [25] found many clusters in the whole San Francisco Bay Area with two large, statistically significant peaks in the East Bay. Further, at the county level, there was statistically significant clustering along the western portion of Alameda County. The researchers then used the city-wide data to identify West Oakland as a TRI source cluster and confirm their hypothesis that West Oakland is an environmental justice site because it has a statistically significant clustered distribution of TRI facilities. Figure 9b shows the location of the TRI clusters calculated with the intensity function, and Figure 9c illustrates that the city-wide cluster is outside of the random Poisson distribution or CSR envelope (represented by the upper and lower solid lines). The researchers go on to integrate social and economic characteristics from the census to explore other area-level demographics that make West Oakland an area for environmental justice concern.

**Figure 10.**
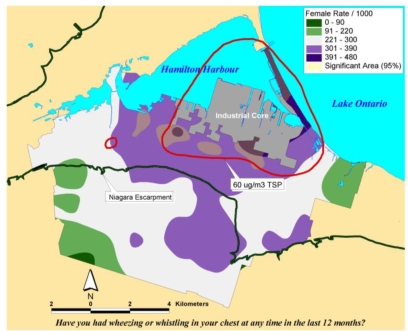
Overlay Map of TSP Exceedance Zone on Interpolated Female Asthma Indicator Rates. Areas within the red isolines indicate zones where the regulatory standard for total suspended particulate matter was exceeded. Areas showing in yellow hatching overlapping with the blue and purple shading indicated rates of asthma symptoms that exceed what would be expected by chance based on a Monte Carlo simulation.

For certain types of disease analysis point pattern analysis can provide useful insights (e.g., the incidence of asthma among young adults 20−44 years old). Data for this example came from a respiratory health survey administered in 1993−94 [17]. Recently developed methods and software are available to interpolate point patterns into continuous surfaces [1]. In addition, it is possible to perform a point pattern analysis with multiple variables using a cross K-function [4]. With these methods, a regular lattice of points or the centroids of some existing lattice such as census tract centroids serves as the vehicle of aggregation. A buffer of a given distance, for example 1.5 km, is drawn around each point in the lattice. Within this buffer there will be a certain number of sampled cases (e.g., young adults 20−44 years old, some of whom will probably have asthma). A rate of asthma is then calculated based on the ratio of cases to the total sample (say, 30 cases over 100 sampled or a rate of 300 per 1,000). The rates are then interpolated by a linear contour, with the result being a continuous surface of disease. Monte Carlo simulation is then used to assess statistical significance. With this simulation, each case is given an equal probability of having asthma and the point pattern is simulated many times (in this case 1,000) to generate a randomized surface. Rates for this surface serve to assess significance. If 950 of the 1,000 simulated surfaces have rates less than the one observed, we can say we are 95% confident that this rate could not have occurred by chance. Figure 10 shows a map for women aged 20−24 years in Hamilton randomly selected as part of a respiratory survey of some 3,300 adults of both sexes in this age group in the city. As is evident on the map, there appears to be an association between higher asthma rates and proximity to a major industrial area that emits particulate air pollution.

In both examples above, we explored “first order” intensity or the tendency of some areas to display a higher density of point cases. Other point pattern analyses like Ripley’s K function seek to assess “second order” effects that measure spatial interaction between the points at various distances [13]. These tests can be useful for testing hypotheses about infectious disease transmission, and as discussed next, more advanced models can include a temporal component.

### Spatial Correlation and Regression

A final type of modeling deals with spatial association or correlation between two or more attribute values at the same location. For example, we may wish to predict mortality rates in given areas with other attribute data such as socioeconomic, lifestyle, and pollution exposure variables. This approach then becomes similar to regression analysis (see, e.g., [19,26]). Predicting health outcomes from environmental exposure while controlling for other known risk factors leads to suggestive evidence of statistical (and potentially causal) associations. When epidemiologic investigations use health data from contiguous or nearby geographic areas, the data may not provide independent estimates of the dependent variable (e.g., relative risk of mortality). If we account for this lack of independence with covariates that are also spatially autocorrelated in a similar way, then bias and underestimation of statistical variability should be reduced because the error terms from such a model tend to be uncorrelated. If areas differ, however, in some unmeasured or unsuspected way that affects mortality, residuals are likely to be autocorrelated [27]. Careful examination and mapping of the residuals can also suggest geographic locations where the model fails to predict mortality accurately, and this may provide clues as to which factors explain some of the variation in mortality or morbidity. When autocorrelation in the residuals cannot be eliminated by adding new variables or changing the specification of the model, other techniques can be employed to avoid bias and inflated significance levels (see [28,29] for detailed conceptual and mathematical expositions of methods for dealing with autocorrelated residuals). Usually these techniques involve either filtering the spatial autocorrelation out of the model beforehand and running the filtered variables through ordinary or weighted least squares [19,30]; alternatively, autocorrelation can be built into the error term of the model, known generally as the “autoregressive” model [11]. The latter model has many forms, but the most common one is called a simultaneous autoregressive (SAR) model.

**Figure 11.**
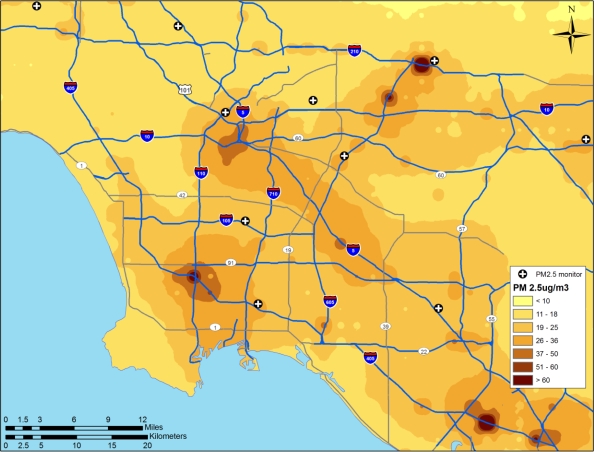
Land use regression prediction surface of particulate matter less than 2.5 microns in diameter (see Moore *et al*. 2007 [31] for more detail on the derivation of the land use regression model).

In another recent study, Jerrett *et al*. [2] investigated the effects of particulate air pollution on mortality in Los Angeles, CA. Figure 11 shows the prediction surface from a land use regression model [31].

The researchers found large significant associations between particulate air pollution and mortality, with especially elevated risks for ischemic heart disease. Risks using this intra-urban exposure assessment were more than two times greater than shown in earlier studies that were based on central monitoring data and used exposure contrasts between cities rather than within them.

**Figure 12.**
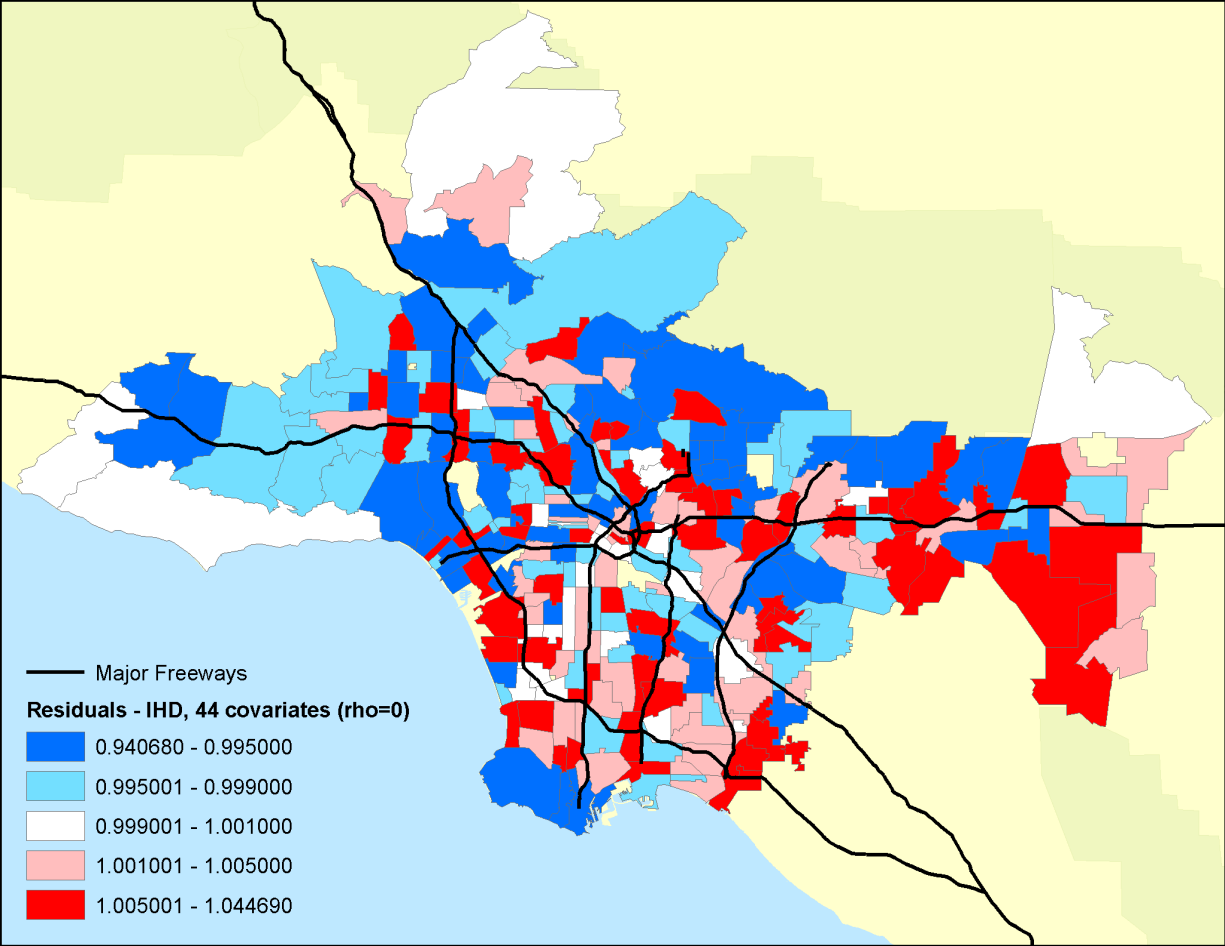
Residual mortality in ZIP code areas after controlling for 44 individual confounders and age, race and sex. Rho represents a spatial autocorrelation term, which was set to zero in this example.

**Figure 13.**
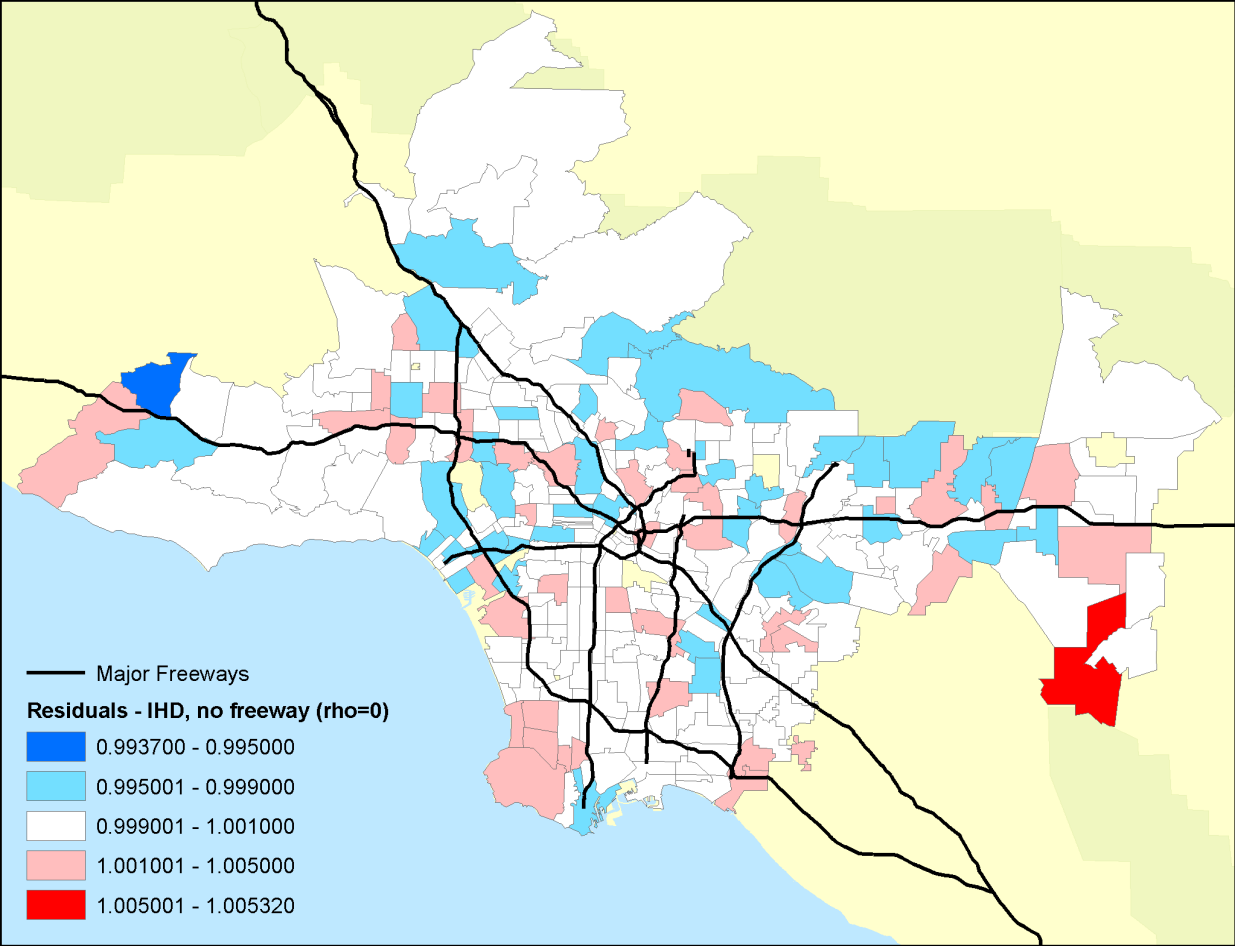
Residual (relative risks of mortality) mortality in ZIP code areas after controlling for 44 individual confounders and age, race and sex with the PM_2.5_ pollution term or autocorrelation term included. Note the decline in the amount of and spatial pattern in the residual mortality.

**Figure 14.**
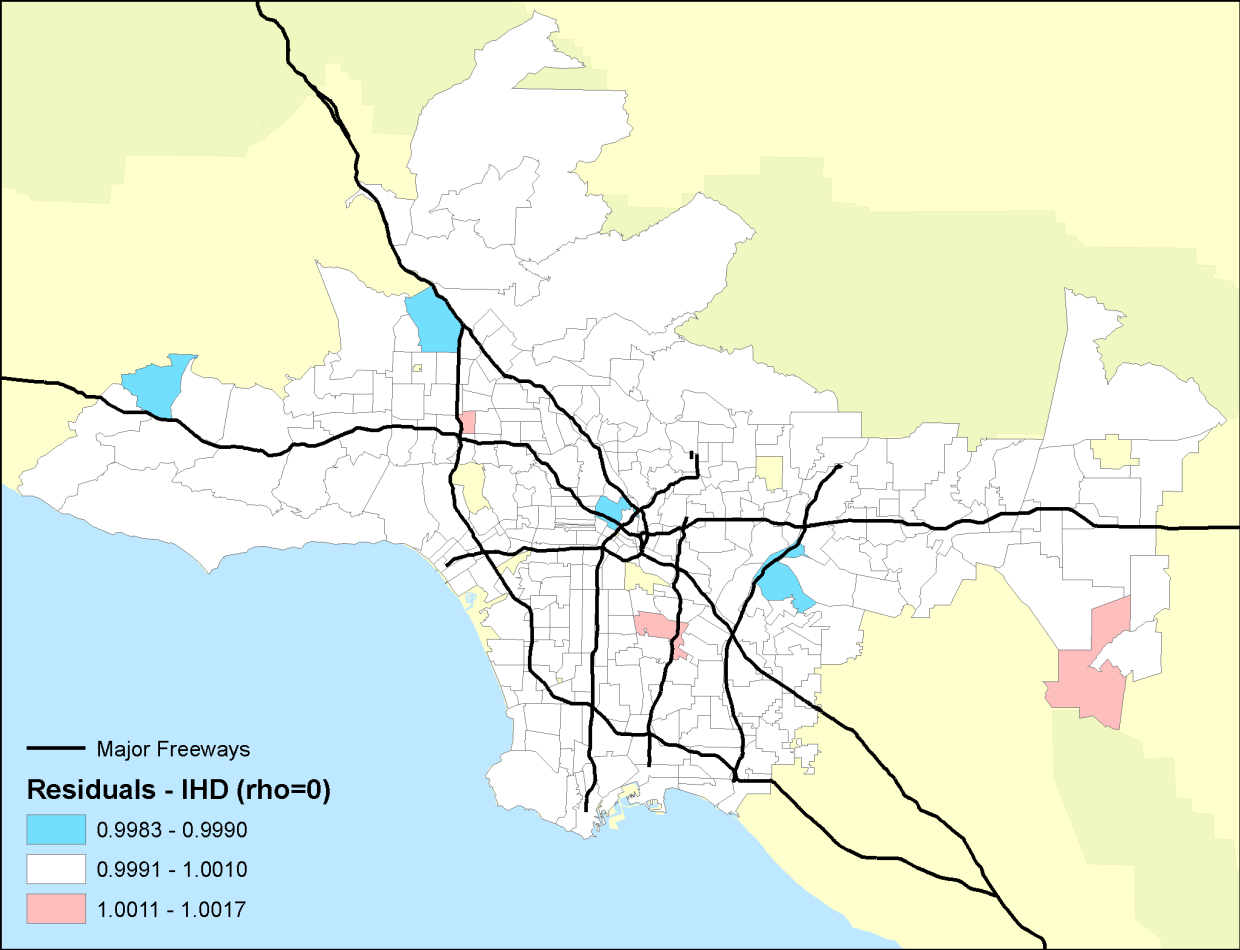
Residual mortality in ZIP code areas after controlling for 44 individual confounders and age, race and sex with the PM_2.5_ pollution and freeway pollution terms included. Note the further decline in the residual mortality and the associated spatial pattern.

Importantly, the researchers were able to examine the residual mortality spatially through multilevel modeling. Figures below show the residual mortality pattern present when only the individual risks are included in the model with no pollution term (Figure 12), and the effect including pollution (Figure 13). There is a substantial reduction in residual mortality when pollution is included in the model. Further analyses in Figure 14 show how inclusion of a term measuring proximity to major freeways further reduced the residual mortality. Statistical tests confirmed significant reductions in residual mortality were associated with pollution, suggesting convincingly that pollution was associated positively with mortality.

These recent methodological advances, with the use of sophisticated Bayesian methods and with multilevel analyses, represent a major new direction in the field. In both instances, confidence in the observed health effects increased substantially with the examination of residual spatial patterns in the data. Removal of these patterns with inclusion of the environmental pollution variables provided stronger evidence that the associations did not occur by chance.

## 4. Emerging Methods

### Mobility and Exposure

Much of the current quantitative work in spatial analysis assigns estimates of exposure to the home address and occasionally to workplace or school locations. Exposure surfaces can be assigned through raster grid cells or as points in a vector-based lattice. The result is a high-resolution estimate of potential ambient exposure across the entire urban area that can be assigned to the subjects’ addresses through the geocoder file that converts alphanumeric street addresses to a longitude-latitude coordinate or equivalent projected coordinate system such as the Universal Transverse Mercator system.

Although useful to use home or work locations, most studies have not assigned exposures based on the “activity space” occupied by individuals. Studies conducted by Kwan [32] indicate high variability in the likely distance away from home during the day. At this stage, much of the research has focused on residential address, but this will have differential levels of accuracy for commuters *vs*. non-commuters, for children being bussed or walking, and for retired *vs*. working individuals. Elgethun *et al*. [33] compared parent diaries to differentially corrected GPS units worn by children 3−5 years old. There was 48% disagreement between the two instruments, with some areas of exposure being significantly underestimated (e.g., time in transit, time outdoors at home). Emerging technologies such as GPS and related activity measures such as accelerometers offer possibilities of reducing such errors in the exposure assignment of individuals in health studies concerned about environment risk.

### Remote Sensing

Remote sensing has emerged as an important innovation in the exposure sciences. Remote sensing can be defined as “the acquisition and measurement of data/information on some property(ies) of a phenomenon, object, or material by a recording device not in physical, intimate contact with the feature(s) under surveillance” [34]. The field encompasses the capture, retrieval, analysis and display of information on surface and atmospheric conductions that is collected using satellite, aircraft or other technologies designed to sense energy, light or optical properties at a distance. Here we review the potential uses of remote sensing for understanding the exposures from traffic pollution under three categories: (1) a means of estimating concentrations of pollutants, potentially generated by traffic, that may associate with health effects; (2) as a direct data input to models used to predict air pollution from land use, traffic, or other ground-level information; and (3) as a means of cross-validation for land or atmospheric data capture by ground or traditional meteorological devices.

### Remote Sensing for Predicting Surface Concentrations

Because routinely collected satellite data capable of measuring parameters that estimate ground level concentrations are generally of coarser resolution than the 500 m distance selected as a guide for traffic impacts [35], we have few identified direct applications of remote sensing to estimating fine-scale variations in traffic pollutants at resolutions relevant to health effects assessment. The Moderate Resolution Imaging Spectroradiometer, which operates from the Terra (EOS AM) and Aqua (EOS PM) satellites [36], currently has capacity to measure aerosol optical thickness (AOT), and when combined with appropriate processing and analysis, to predict particle concentrations in the troposphere. Some of the better retrievals and predictive models have been for relatively large areas on 1 × 1 degree grids, which translate into about 110 km resolution at the Equator. The minimum grid size available currently from MODIS is 10 × 10 km grids, with global coverage on a two day cycle. Liu *et al*. [37] demonstrated a method for retrieving and reprocessing the MODIS images to a 1 km resolution, however, this method needs further development before being employed in epidemiological studies. Based on a three day comparison against 11−14 ground level measurements of PM_10_, correlations ranged from 0.55−0.86. While the predicted values are for areas slightly larger than the near-source influence zone, further refinements to scales useful to assessing health effects of traffic appear likely.

The Multi-angle Imaging SpectroRadiometer (MISR) is another space-based instrument capable of estimating AOT. This instrument has a minimum grid size of 17.6 × 17.6 km, and temporal coverage of the Earth every nine days [38]. Recent studies have utilized MISR to predict PM_10_ surface concentrations within Beijing, China [39]. The authors found moderately high correlations between measured concentrations and MISR predictions in the Fall, Winter and Spring (r ranging from 0.59 to 0.72), but a weaker correlation in summer (r = 0.32). Although the MISR predictions characterized the spatial pattern of AOT fairly well over the broad metropolitan area of Beijing, the authors noted that the minimum grid size of 17.6 km may be insufficient for assessing spatial variation in areas with high levels of heterogeneity in particle concentrations within the city.

Special studies using Light Detection and Ranging (LiDAR) have been used to augment other meteorological and ground-level data for understanding spatial and temporal dimensions of aerosols [40]. In theory, LiDAR may produce 1 m resolution images, but it has limitations in terms of oversensitivity to coarse particles (Brook, personal communication 2007) in estimating particle concentrations. Future studies using LiDAR may allow for highly refined estimates of exposure from traffic pollution.

### Remote Sensing as Data Input

Increasingly land cover information is derived partly or wholly from remotely sensed imagery. For example, as mentioned earlier, the US Multi-Resolution Land Characteristics Consortium of federal agencies has purchased and processed Landsat 7 images to classify land cover for the National Land Cover Database, which encompasses the entire US [41]. This database provides land use data in a raster grid cell format at 30 m resolution. Earlier versions of this land cover data were used to calibrate a land use regression model in New York City for predicting small area variations in PM_2.5_ [42] and similar information is available at the national scale, which will enable large-area models of many cities to be calibrated where the pollution monitoring data exist or are collected for special studies.

**Figure 15.**
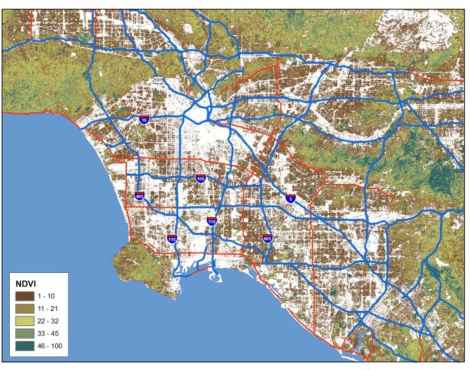
Normalized Difference Vegetation Index for the Los Angeles Metropolitan Area based on Landsat Imagery. Compare to Figure 13 to see the similarities between areas of high pollution and low vegetation.

Processed images may also supply useful information as input to exposure models. As an example, the normalized difference vegetation index (NDVI) can be used to derive estimates of vegetative cover (see figure 15). These have been used as predictors in land use regression models, and because the green cover supplies an alternate estimate of those areas likely to have fewer mobile sources, future applications of the NDVI and other processed images may serve as important data inputs to traffic exposure assessments.

### Remote Sensing for Cross-validation

Many of the current exposure models used to predict pollutant concentrations at a fine scale utilize ground-based information on pollutant concentrations, land use and traffic. In some instances, the geographic accuracy of these ground data may be of variable or questionable quality. Remotely sensed imagery of high resolution can be used as cross-validation against which to compare these ground data. Some examples include the location of pollution monitoring stations operated by government entities. Although increasingly these sites are marked with GPS coordinates, some error in the GPS coordinates can occur and those that rely on coordinates assigned by paper maps may have large errors. Digital orthophotos or high resolution images from IKONOS or QuickBird images, at 1−5 m resolution, can increase the spatial accuracy of the data used as input to land use regressions (e.g., [42]). Similar comparisons can be done with land use classifications and with road networks. The advent of Google Earth and its extensions has made such cross-validation more accessible for many researchers, and reductions in spatial errors have probably increased prediction accuracy of ground level concentrations.

## 5. Policy Implications

Understanding the interface between scientific research and policy action is a complex and multifaceted undertaking. Prevention policies designed to protect public health usually involve the knowledge base, political will to act, and social strategy to accomplish change [43]. Undoubtedly the knowledge base plays a critical role in stimulating and supporting preventive actions to protect public health. The specific contribution, however, in each instance remains difficult to assess. We have selected three illustrations, each with some level of evidence, to demonstrate how the scientific knowledge base, specifically relying on GIS, influences public health prevention policies.

Some studies have had direct impact on policy. For example, the aforementioned study by Jerrett *et al*. [44] is now being used by the EPA in the review of national ambient air quality standards for PM_2.5_. In addition, this paper was cited in the health burden assessment for the California Air Resources Board. Also, this study was cited by the majority of experts as part of a U.S. EPA expert elicitation on the causal effects of PM_2.5_ on mortality as one of the most influential studies in determining whether a causal relationship existed between PM_2.5_ exposure and mortality. Along with three other prominent studies, the Jerrett *et al*. study was used to assess median effect estimates of PM_2.5_ exposure on mortality [45]. The expert elicitation aimed to inform the EPA on the benefits of its air quality regulations, and specifically on the decrease in mortality rates that could be achieved with decreased PM_2.5_ exposure. This study therefore provides an example of direct linkage to and influence over public health protection policy that relied on a study using GIS and spatial modeling.

The Office of Environmental Health Hazard Assessment (OEHHA) in California has formed a working group with the California Integrated Waste Management Board to assess cumulative environmental impacts and make policy recommendations in accordance with the Cal EPA Environmental Justice Action Plan. Members of this group titled the Cumulative Impacts and Precautionary Approaches (CIPA) Work Group come from industry, academia, and environmental and community groups to collaborate and develop feasible solutions to minimize the effect of adverse environmental impacts. Moreover, environmental justice arguments are being heard in the California legislature with the passage of Assembly Bill (AB) 32, which, as a part of the Global Warming Solutions Act, requires California to reduce greenhouse gas emissions to 1990 levels by 2020. This bill specifically mandates that an Environmental Justice Advisory Committee convene and advise the California Air Resources Board on the development of the planning and implementation of AB 32. Although direct linkages to specific studies are hard to determine, the works of Rachel Morello-Frosch and Jesdale appear to have influenced the consideration of cumulative effects and environmental justice in California because both these scholars are now on the academic partner’s team of CIPA.

## 6. Discussion of GIS Methods and Limitations for Future Public Health Research

This paper has reviewed concepts and methods of spatial analysis used in spatial epidemiology and public health research. Examples from published and ongoing studies served to illustrate the strengths and weaknesses of different types of spatial analysis. We have supplied a reasonably complete summary of the field, but have omitted some point pattern and multivariate methods. For example, principal components analysis may be used to characterize neighborhoods by extracting closely related components of variables describing the social, economic, and demographic characteristics of neighborhoods. The component scores can be mapped and local autocorrelation statistics can be applied to assess hot spots of low socioeconomic status or other areas likely to experience poor health [18]. Information from these analyses can be used to target health surveys or public health messages.

Through this review, we have underscored the key limitations of each method and approach. Other perennial issues related to spatial analysis in a health context deserve mention. First is the ecological fallacy. In deriving group rates for display and analysis in chloropleth form, aggregation from the individual to the spatial unit can lead to incorrect inferences about individuals (referred to as the “cross-level” bias). This issue has been examined in many studies, and while a thorough review is beyond the intent of this paper, ecologic bias may lead to incorrect inference about associations between risk factors and individual health [46]. This may not present a problem when interest lies in assessing determinants of population health on a geographic level. If the research focuses on population health relationships, analysts must then be weary of another aggregation issue, referred to as the “modifiable areal unit problem.” This problem arises due to the uncertainty induced by the aggregation process. Observed spatial patterns might be a function of the zones chosen for analysis rather than the underlying spatial pattern. In other words, spatially aggregated data display higher levels of uncertainty than the individual data on which those aggregations are based, and observed patterns may result from artifacts of aggregation [13]. Some analysts suggest that the smallest available unit of analysis should be used unless prior evidence indicates larger units will reveal more about the health effect in question [4].

Relying on small units can lead to low counts of health data and subsequently unreliable rates, especially for rare diseases and events such as mortality. Various techniques have evolved for dealing with the “small numbers problem” in disease mapping [47]. Both frequentist and Bayesian methods are used for dealing with small counts in some of the spatial units. Most of these methods convey those spatial units that have small counts that would produce unreliable visualization or inference. For example, some analysts have suggested using Bayesian adjustment procedures to produce rates that balance observed rates and some global or local mean value [48], with the latter receiving greater weight in the calculation when underlying population and event counts are small. Such methods have been criticized for distorting inherent spatial patterns [49]. Other methods involve weighting for estimation uncertainty, similar to the ones used in our spatial regression models presented earlier. None of these methods compensates completely for a lack of information due to small counts. An unavoidable tension always exists between minimizing aggregation bias and maintaining counts large enough to ensure reliable rates.

Some of the point pattern techniques discussed earlier rely on simulated data and Monte Carlo distributions to overcome the problem of small counts by using data from larger areas created by buffers that circle a point representing a health outcome or the centroid of an existing administrative unit such as a census tract [50]. As noted, most of these models lack the ability to produce rates that control for confounders such as age, which is a considerable limitation. The question also arises as to what the rates mean when they are brought back into administrative units (e.g., census tracts) because data from outside the tract has been used to compute the rate.

Finally, in most spatial analyses, controlling simultaneously for all known risk factors is problematic, and analysts may have to rely on both temporal and spatial methods. This is especially true for acute exposures that elicit a health response within a short time frame. For example, Poisson regressions of mortality counts on air pollution and weather variables, with appropriate adjustment for serial autocorrelation, build in automatic control for confounding because individuals experiencing health effects are unlikely to change their job, lifestyle, diet, and other risk factors within a short period of 1−3 days [51]. Investigating the same association between air pollution and mortality through spatial analysis would require control for many potential risk factors [17], and operationalizing such models without a high degree of collinearity is a difficult if not impossible task [26]. A thorough spatial analysis of the same relationships may still uncover useful information, including exposure mismeasurement within metropolitan areas that may not be apparent through the time series, the effect of specific confounders, and the influence of chronic exposure. Methods such as Cox regressions, GAMs, and Bayesian modeling can incorporate time and space as well as individual and ecologic effects. These and similar multilevel, multidimensional models may reveal insights unavailable from methods that focus on any one level or dimension. Seen from this perspective, both temporal and spatial methods assist researchers with triangulating on the etiology of disease. Thus, despite the numerous epistemological, methodological and data challenges to spatial methods, many environmental health investigations can benefit from the careful application of the spatial analysis.

Given the potential of these methods, what are their prospects for future use in environmental health research? We will probably see further proliferation of spatial analysis as the methods become more familiar to researchers outside of medical geography and spatial epidemiology. The largest challenge to the expanded use of GIS and allied methods for health surveillance relates to data availability, consistency, and cost. In the United States, the myriad of private medical care suppliers will probably make the task of developing national level data capable of supporting spatial analysis even more difficult. Thus, while the knowledge and the technology are available to utilize spatial analysis in Public Health, the institutional structures for data collection, management, and dissemination are lagging. Until these structures are developed and put in place, spatial analysis will remain in the realm of a specialized approach for specific studies where data are available. While the development of “infostructure” may seem costly, the expense amounts to a rounding error on the expenditures currently made in traditional medical care.

## 7. Conclusions

Through this review some central conceptual issues and trends have emerged. In examining the trends, there has been a remarkable growth in the use of advanced spatial modeling that appears an essential component of spatial epidemiology and public health. Use of GIS and spatial analysis is now commonplace in many research projects and health departments, oftentimes not involving traditional health geographers.

On the assessment of health risks, the methodological advent of multilevel models and substantive idea of contextual influences on health have done much to increase the sophistication and insights into how environmental risks are both conditioned and confounded by numerous social and neighborhood factors. The use of multilevel models has elevated insights into health risks—in some of the more advanced models, the spatial approach has lead to much higher confidences in the empiric results and the demand for this kind of modeling in a field always at the interface between science and policy appears likely to grow.

Other future trends are also apparent. GPS systems and activity monitors have given researchers capacity to move beyond relatively static geographies of risk, with exposures assigned largely to the home address, to characterize mobility and activity while in the exposure space or what Hägerstrand called the “hazard fields”. Interesting and counter intuitive findings are emerging from such studies. For example, Briggs *et al*. [52] recently demonstrated that exposures to air pollution were higher for children who walked to school in London, England, than for children who were driven in automobiles. While the promise of capturing a time-geography of risk has not yet been fully realized, it is much closer to reality now than ever.

Although still in its infancy, remote sensing holds promise for studying environmental exposures and even for characterizing susceptibilities, particularly in poorer regions that may lack digitized mapping data. Remote sensing as presented through Google Earth has also awakened the geographic imagination in ways that go beyond the traditional academy and places where health geography is typically practiced. Numerous sites have now used Google Earth to map environmental exposures and risks. Combined with more systematic efforts of web-based mapping [53], Google Earth and similar applications appear destined to have a major influence on the field of public health sciences.

This paper has reviewed the rationale for GIS and spatial analysis in environmental and public health research, with an emphasis on earlier arguments by Mayer [1] and on the data issues that often limit environmental epidemiology and public health. From there the paper adapted a “Geography of Risk” framework emphasizing that risks to human health often result from the overlaps among individual susceptibility, exposure to environmental toxins and (mal)adaptation to those exposures or the stresses they cause. Recent trends in the field were examined with a literature review covering 20052–008. Through this review, progression toward more methodologically sophisticated methods is evident. GIS and allied methods are now essential components in the larger fields of epidemiology and public health. This influence is evident with growing use of the scientific outputs for informing public health prevention policies and practices.

## Figures and Tables

**Figure 8a.**
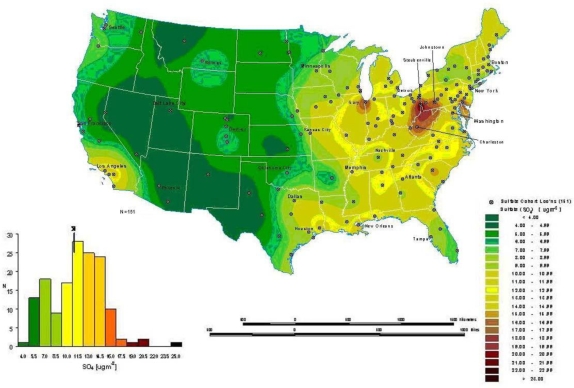
Modeled Mean Concentrations of Ambient Sulfates in the ACS Study.

**Figure 8b.**
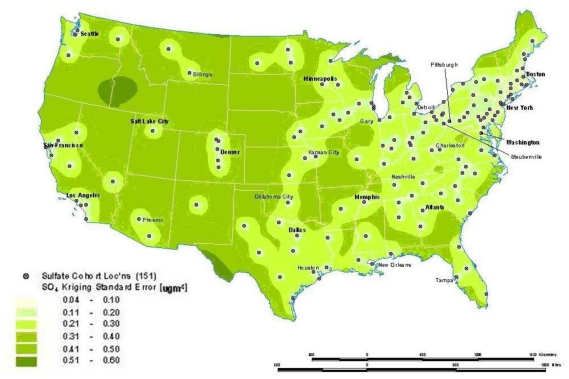
Standard Estimation Error Associated with Interpolated Concentrations of Ambient Sulfate Using Kriging.
